# How, Why and When Tsallis Statistical Mechanics Provides Precise Descriptions of Natural Phenomena

**DOI:** 10.3390/e24121761

**Published:** 2022-12-01

**Authors:** Alberto Robledo, Carlos Velarde

**Affiliations:** 1 Instituto de Física and Centro de Ciencias de la Complejidad, Universidad Nacional Autónoma de México, Mexico City 04510, Mexico; 2 Instituto de Investigaciones en Matemáticas Aplicadas y en Sistemas, Universidad Nacional Autónoma de México, Mexico City 04510, Mexico

**Keywords:** Tsallis entropy, Landau–Ginzburg equation, chaos transitions, renormalization group fixed-point maps, complex systems

## Abstract

The limit of validity of ordinary statistical mechanics and the pertinence of Tsallis statistics beyond it is explained considering the most probable evolution of complex systems processes. To this purpose we employ a dissipative Landau–Ginzburg kinetic equation that becomes a generic one-dimensional nonlinear iteration map for discrete time. We focus on the Renormalization Group (RG) fixed-point maps for the three routes to chaos. We show that all fixed-point maps and their trajectories have analytic closed-form expressions, not only (as known) for the intermittency route to chaos but also for the period-doubling and the quasiperiodic routes. These expressions have the form of *q*-exponentials, while the kinetic equation’s Lyapunov function becomes the Tsallis entropy. That is, all processes described by the evolution of the fixed-point trajectories are accompanied by the monotonic progress of the Tsallis entropy. In all cases the action of the fixed-point map attractor imposes a severe impediment to access the system’s built-in configurations, leaving only a subset of vanishing measure available. Only those attractors that remain chaotic have ineffective configuration set reduction and display ordinary statistical mechanics. Finally, we provide a brief description of complex system research subjects that illustrates the applicability of our approach.

## 1. Introduction

In the late eighties, over thirty years ago, Constantino Tsallis initiated [1] a novel research path in statistical physics, different in character with all other pioneering efforts of that time in that it entered the topic of the theoretical structure of statistical mechanics. Tsallis explored the formal changes in the statistical mechanical and thermodynamic formalism by replacing the customary entropy expression of Boltzmann and Gibbs (or of Shannon) by a mathematical generalization of it [2], that here, succinctly, we refer to as the substitution of the ordinary logarithmic function by a generalized logarithm, a one-scalar deformation of it known as the *q*-logarithm,

(1) $$\ln_{q}\left(x\right)\equiv \left[x^{1-q}-1\right]/\left(1-q\right),$$

where *q* is a real number. The ordinary logarithm is recovered when q=1. While many statistical-mechanical relationships appeared unchanged or their variants were not difficult to obtain after the entropy expression replacement [3], other aspects posed difficulties and led to controversies [4]. These developments produced an enthusiastic response that translated into many studies and related publications [3], but also to skepticism [5]. Implicit to these investigations lies the most fundamental issue, the existence of a limit of validity for ordinary statistical mechanics and the reason for its occurrence. Parallel to these activities the field of study of complex systems grew and captured the interest of many scientists, specially those in the area of statistical physics. This field provides a wide family of phenomena that suggests possible candidates for the applicability of generalizations of statistical mechanics.

Interestingly, features at the transitions to chaos displayed by dissipative nonlinear systems were found to follow, quantitatively, *q*-logarithmic laws, or equivalently, laws that follow its inverse function, the *q*-exponential function,

(2) $$\exp_{q}\left(x\right)\equiv {\left[1+\left(1-q\right)x\right]}^{1/1-q}.$$

In other words, at these borderline circumstances a replacement takes place of the pair of ordinary logarithm and exponential functions upon which ordinary statistical mechanics is expressed [6,7]. A generalization of the Pesin identity (that expresses the equality between a *q*-generalized Lyapunov exponent with the Tsallis entropy growth rate) was shown to hold at the period-doubling accumulation points [6,8] of quadratic maps. Moreover, the analytical closed-form Renormalization Group (RG) fixed-point map originally derived by Hu and Rudnick [9], was identified (except for a necessary linear factor) as a *q*-exponential, as well as its presence in all its possible trajectories [6,7,10]. Likewise, the transition to chaos at the golden mean in the circle map was shown to comply with these features [7,11]. Thus, the transitions to chaos along the three known routes, period doubling, intermittency and quasiperiodicity, were seen to obey the basic mathematical elements of Tsallis statistics, properties easily corroborated via the consideration of the well-known, and simple one-dimensional iteration maps. See Ref. [7] for a detailed discussion. For early numerical evidence of *q*-exponential behavior at the period-doubling transition to chaos see Refs. [12,13]. For initial numerical indications of the *q*-Pesin identity see Refs. [14,15]. For first numerical observations of *q*-exponential conduct at the golden mean transition to chaos in the circle map see Ref. [16].

Furthermore, over the last two decades a number of central problems for complex systems were modelled around the mentioned RG fixed-point maps that represent the three routes to chaos, the analysis of which led to both fresh insights and correct predictions with explicit presence of the Tsallis entropy, and their associated distributions or Lyapunov exponents [6,7,17,18]. Amongst condensed matter physics problems there are: dominant fluctuations in critical phenomena, dynamics of glass formation, and wave-scattering localization transitions. Concerning key, shared, complex systems questions: thermodynamic stability of self-organization (including critical self-organization) [19], equivalence of paradigms (edge of chaos and criticality) [20], and crossover from multifractal to normal stationary distributions. Within mainstream topics: Zipf law and other rank distribution laws [7], routes to chaos in game theory, and scale invariance of networks from time series.

From the above we can conclude that, if a formal analytical bridge is provided from a well-defined statistical-mechanical point of departure to the RG fixed-point maps, and to the properties of their attractors, a firm basis will be delivered to Tsallis statistics, a clarification of its range of appropriateness, and a wide class of subjects of application within complex system phenomena. Here we offer this connection. Our starting point is the formalism employed to describe the kinetics of phase change in condensed matter in its dissipative version given by the Landau–Ginzburg equation [21,22]. This approach has proved to be a valuable tool for the description of nucleation, spinodal decomposition, and soliton phase growth [23]. Next, we particularize this procedure to discrete time evolution to obtain the RG fixed-point map for the tangent bifurcation [9]. We justify the appropriateness of discrete time (or when applicable that of its equivalent model variable) for complex system phenomena. See next Section 2. Then, we show that the trajectories of the RG fixed-point maps for the period doubling and the quasiperiodicity routes to chaos (that have to date only been known numerically via approximations of the power series representations of their fixed-point maps [24]) can in each case be obtained analytically in a closed form equivalent to the *q*-exponential expression for the intermittency route [9]. As we explain, this requires specific sets of iteration time changes of variable. That is, all the properties of model applications of the RG fixed-point maps obey analytic expressions precisely linked to the *q*-deformed exponential and logarithmic functions for the distributions and entropies of Tsallis statistics. See Section 3. After this, we consider the limit of validity of ordinary statistical mechanics and the subsequent incidence of Tsallis statistics. These circumstances are a consequence of the effect of the RG fixed-point maps attractors in reducing drastically the initial phase space, or in terms of the phenomena modelled by them, in a severe hindrance to access initially available configurations. The vanishing measure of the remaining set of configurations is quantitatively measured by a contraction dimension related to the deformation paramenter *q* [7]. See Section 4. Finally, we give a brief description of several research line topics (already quoted above) in the field of complex systems with reference to their relationships with Tsallis statistics [18]. See Section 5. We end with a brief Summary and Discussion.

A word about the scope of the results presented below. Our description focuses on the discrete-time version of first-order differential equations, standard nonlinear iteration maps that are then studied at transitions to chaos; i.e., low-dimensional nonlinear dynamics. In statistical-mechanical terms the properties obtained correspond to the most probable evolution of processes undergone by systems composed of large numbers of degrees of freedom, just as the usual Landau–Ginzburg kinetic equation does for macroscopic collective processes observed in condensed matter systems. Furthermore, the emphasis on the RG fixed-point maps captures the main features of large families of maps (or Landau–Ginzburg difference equations). Besides the known RG *q*-exponential trajectories we show that the Tsallis entropy is the Lyapunov function of the RG Landau–Ginzburg equation.

## 2. Most Probable Evolution of Fluctuations

Nonlinear processes in systems with many degrees of freedom, including phase change in condensed matter, proceed via initial fluctuations in the starting state. Many fluctuations are transitory and leave the state unchanged, but some, sufficiently strong in some required sense, carry the system into a different one. The most probable time evolution of fluctuations in systems composed of many degrees of freedom can be described via rate equations. A macroscopic rate equation for dominant fluctuations was derived a long time ago by Metiu, Kitahara and Ross (MKR) [21,22]. These authors had shown that averaging a microscopic master equation, containing detailed information on the individual degrees of freedom, over intermediate time scale intervals led to a final macroscopic rate equation where this information manifests only through changes in a generalized thermodynamic potential Ω (of the type used in density functional theories [25]). Below we relate the potential Ω to the Tsallis entropy when we reduce the discussion to a class of dissipative nonlinear discrete-time systems, i.e., iterated maps. The MKR equation reads [21,22]

(3) $$\frac{\partial \rho (r,t)}{\partial t}=-u\left[1-\exp(1-\lambda^{2}\nabla^{2}/2)\right]\frac{\delta \Omega }{\delta \rho (r)}{|}_{\rho (r,t)},$$

where ρ is the quantity of interest to be described, such as a fluid density, *r* is spatial position, *t* is time, and Ω is a Lyapunov function [26], here a generalized thermodynamic potential. The functional derivative δΩ/δρ is the main ingredient of the rate equation driving force, the right-hand-side of the equation, it is a generalized thermodynamic field. In the case of a fluid it is a generalized chemical potential, where Ω is a generalized grand potential free energy (both space and time dependent unlike the final equilibrium ones). Furthermore, in the case of a fluid, the parameter λ is the mean distance transversed by a particle in the time interval (0,t). Equation (3) reduces, respectively, when the variations of ρ are small or large with respect to λ to the conservative Cahn–Hilliard equation [21,22],

(4) $$\frac{\partial \rho (r,t)}{\partial t}=u^{\prime }\nabla^{2}\frac{\delta \Omega }{\delta \rho (r)}{|}_{\rho (r,t)},$$

and to the dissipative Landau–Ginzburg equation [21,22],

(5) $$\frac{\partial \rho (r,t)}{\partial t}=-u\frac{\delta \Omega }{\delta \rho (r)}{|}_{\rho (r,t)}.$$

Above *u* and $u^{\prime }$ are positive constants. These equations have been successfully used in describing nucleation, spinodal decomposition and interface motion [23]. The most important feature of the Lyapunov function, the generalized thermodynamic potential, is that it varies monotonically with time along the solution of the rate equation. For the particular case of the fluid where Ω is a generalized free energy we have

(6) $$\frac{\partial \Omega [\rho (r,t)]}{\partial t}\leq 0.$$

In what follows we make use only of the Landau–Ginzburg equation for which the driving force of the rate equation is simply proportional to the functional derivative of the Lyapunov function.

Discrete time (or its equivalent variable in specific model studies) description is the appropriate choice for many complex systems phenomena. The reasons for this may be circumstantial, like in the study of the magnetization of a spin system via consecutive updates of Monte Carlo generated configurations. In other cases it is naturally required, like in the empirical Zipf law and other ranked data distributions. Other instances are the spread of population contagions, the evolutionary tree of life, or the sizes of animal groups in collective motion, etc. In anticipation of the link we present below between rate equations in statistical mechanics and dissipative iteration maps in nonlinear dynamics, we present a discrete-time version for the Landau–Ginzburg equation. This is

(7) $$\frac{\rho (r,t+1)-\rho (r,t)}{(t+1)-t}=-u\frac{\delta \Omega }{\delta \rho (r)}{|}_{\rho (r,t)},$$

or, the iterated map

(8) $$\rho \left(r,t+1\right)=\rho \left(r,t\right)-u\frac{\delta \Omega }{\delta \rho (r)}{|}_{\rho (r,t)}.$$

Changing subject, we recall now the essentials of the derivation of the RG fixed-point map $f^{*}\left(x\right)$ associated with the intermittency route to chaos [9,24]. In the neighborhood of a map at tangency with the identity line at x=0 with nonlinearity *z* we have

(9) $$f\left(x\right)=x+{u|x|}^{z}+\ldots ,z>1.$$

The definition of the RG fixed-point map $f^{*}\left(x\right)$ is to satisfy the condition,

(10) $$f^{*}\left(x\right)=\gamma f^{*}\left(f^{*}\left(x/\gamma \right)\right),$$

for some specific form of the scaling factor γ and to reproduce Equation (9) for small *x*. The expression for the RG fixed-point map $f^{*}\left(x\right)$ is [7,9]

(11) $$f^{*}\left(x\right)=x\exp_{z}{\left(u|x\right|}^{z-1}\mathrm{sgn}\left(x\right)),\;\gamma =2^{1/(z-1)},$$

while the expression for all of its trajectories is [7]

(12) $$x_{\tau }=x_{0}\exp_{z}(\mathrm{sgn}\left(x_{0}\right)|x_{0}{|}^{z-1}u\tau ),\tau =0,1,2,\ldots$$

The RG fixed-point map and its trajectories at the tangent bifurcation, Equations (9), (11) and (12), have a precise probabilistic analogue, an uncommon equivalence between a deterministic system and a stochastic one. This is explained in detail in Refs. [27,28]. The random variable in the stochastic process is −x in f(x) of Equation (9) for x<−1, the parent or source distribution is proportional to ${\left|x\right|}^{-z}$, the cumulative distribution is the sum of trajectory positions $|x_{\tau }|$ and the quantile is $|x_{\tau }|$ itself. The parent distribution is a power law while the quantile is a *q*-exponential [27,28]. The quantiles are not probability distributions, they are cut points dividing the range of a probability distribution into continuous intervals with equal probabilities, or dividing the observations in a sample in the same way. A uniform distribution can be assigned to each quantile interval (that in statistical-mechanical terms turns out to correspond to the distribution of a microcanonical ensemble), and another distribution can be assigned to the entire set of quantile intervals (that in statistical-mechanical terms turns out to correspond to the distribution of a canonical ensemble).

Making use of the above we consider trajectories of $f^{*}\left(x\right)$ initiated at $x_{0}<-1$ and stopped at $x_{\tau_{\max}}\leq -1$, so that the reciprocals of $|x_{\tau }|>1$ provide uniformly-distributed probabilities $p_{i}^{\left(t\right)}=p^{\left(t\right)}=1/\left|x_{t}\right|,i=0,\ldots ,t$, where *t* is fixed. The value of *t* relates to the iteration τ according to $t=\tau_{\max}-\tau$. There is one set of t+1 equal probabilities for each value of *t*, $0\leq t\leq t_{\max}$, where t=0 corresponds to $\tau_{\max}$ and $t_{\max}$ to τ=0. That is, for each value of *t* the number $|x_{t}|$ is divided into t+1 equal parts that form the t+1 values of the uniform distribution. The values of these probabilities decrease while the number of the set of equally-probable outcomes, i=0,…,t, increases as *t* increases. The discrete-time Landau–Ginzburg equation, for the particular case of spatial uniformity, for the sets of probabilities $p^{\left(t\right)}$ is

(13) $$p^{\left(t+1\right)}=p^{\left(t\right)}-u\frac{\partial \Omega }{\partial p}{|}_{p^{\left(t\right)}},$$

where

(14) $$\frac{\partial \Omega }{\partial p}{|}_{p^{\left(t\right)}}=-{\left[p^{\left(t\right)}\right]}^{-q},q=-z,$$

and the Lyapunov function is

(15) $$\Omega \left(t\right)=\log_{Q}\left(\right|x_{t}\left|\right),$$

where Q=2−q.

We arrived at the most interesting result in this Section: the Lyapunov function in Equation (15) can be identified with the Tsallis entropy

(16) $$S_{Q}=-\sum_{i=0}^{t}p_{i}\ln_{Q}p_{i},$$

for a distribution of equal probabilities. This entropy is extensive where system size *N* is measured by trajectory duration $t_{\max}=N$. The canonical distribution composed from the set of microcanonical distributions $p^{\left(t\right)}$ is the *q*-exponential

(17) $$p^{\left(t\right)}=p^{\left(0\right)}\exp_{q}\left(-{\left[p^{\left(0\right)}\right]}^{1-q}ut\right),\;t=0,1,2,\ldots$$

**Figure 1:**
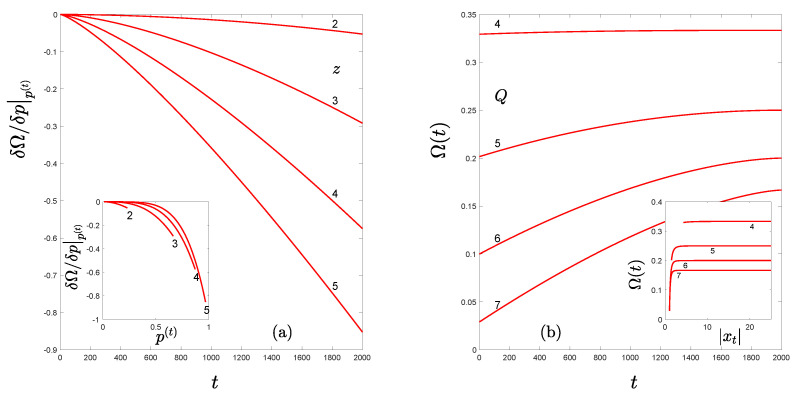
(**a**) The driving force $\frac{\partial \Omega }{\partial p}{|}_{p^{\left(t\right)}}$ in Equation (14) for the kinetic Equation (13) as a function of iteration time *t*. The inset shows the same quantity as a function of the probability $p^{\left(t\right)}$. Different values of the nonlinearity *z* of the RG fixed-point map are indicated. (**b**) The Lyapunov function Ω(t) in Equation (15) (Tsallis entropy) as a function of the iteration time *t*. The inset shows the same quantity as a function of the absolute value of trajectory positions $|x_{t}|$. Different values of the Tsallis entropy index Q=2+z are indicated.

The difference between the indices in the entropy and in the distribution, Q=2−q and *q*, respectively, will be explained below. See also Ref. [7]. Notice that, unlike the fluid case described before, the Lyapunov function Ω is now a generalized Massieu potential [29] that grows monotonically,

(18) $$\frac{\partial \Omega [p^{\left(t\right)}]}{\partial t}\geq 0,$$

to a maximum. See Figure 1.

## 3. RG Fixed-Point Maps for Other Routes to Chaos

The Renormalization Group is a statistical-mechanical technique designed for the study of system states or processes characterized by scale invariance, and therefore expressed by power law behavior. A particular class of scale invariant system states are the transitions to chaos in low-dimensional dissipative nonlinear maps. For these cases the natural RG transformation is functional composition, and the condition to be satisfied by the RG fixed points is exceptionally uncomplicated. This we have illustrated for the tangent bifurcation in Equation (10), the point at which the transition out of chaos via intermittency takes place [24]. The same RG fixed-point map condition, Equation (10), applies at the transition to chaos via the period doubling route. This was originally employed by Feigenbaum [24] to study the accumulation point of the period-doubling cascade of bifurcations, the transition to chaos present in all unimodal maps. The case of the third route to chaos, via quasiperiodicity, occurring for an infinite set of irrational numbers in the circle maps, requires a different (a step more complicated) RG fixed-point map condition [24],

(19) $$f^{*}\left(x\right)=\gamma f^{*}\left(\gamma f^{*}\left(x/\gamma^{2}\right)\right).$$

Chronologically, the first RG fixed-point map to be determined was that, for the period-doubling route, no analytical closed-form expression was accessible, but the properties of $f^{*}\left(x\right)$ were characterized by means of a precise power-law series expansion of the resultant trascendental function, together with the value of its universal constant scaling factor γ for the quadratic case [24]. The second RG fixed-point map to be resolved and analyzed was that for the quasiperiodic route to chaos leading to a similar situation with regards to mathematical expressions and universal constants [24]. A prominent role was played by the special golden-mean route to chaos [24]. The last RG fixed-point to be investigated was that for the tangent bifurcation [24], and as a difference with the previous two, an analytical closed-form expression was found [9,24], that which we have already detailed above and, as it is our purpose, reveal its link with the incidence of Tsallis statistics.

Our aim now is to show that all the RG fixed-point maps for the three routes to chaos share in fact the analytical expression Equation (11) provided some specific additional initial position and iteration time rules are incorporated to obtain their more complex trajectories from Equation (12). This surprising mathematical fact, detailed below, that all of the trajectories from the RG fixed-point maps can be written in the closed-form given by Equation (12), together with specific sets of prescriptions for initial positions and changes in the time iteration variable, is an important advance in the nonlinear dynamical topic of RG fixed-point maps, but it is also crucial for our objectives here. The immediate consequence is that all trajectories from the RG fixed-point maps can be associated with *q*-exponential probabilities and with Lyapunov functions relatable to the Tsallis entropy expression.

Before we proceed it is pertinent to reiterate that all of the trajectories of the RG fixed-point map for the intermittency route can be written in the closed form Equation (12). Actually any trajectory of this map can be transformed into any other via rescaling of *z*, *u* and/or $x_{0}$. This can be done in more than one way; in particular a change in the initial position can be reproduced also by a change in *u*. Indeed, the factor $x_{0}^{z-1}$ can always be absorbed into the parameter *u*. The duration of a trajectory can also be adjusted via a modification of either the counting iteration variable τ or the parameter *u*.

**Figure 2:**
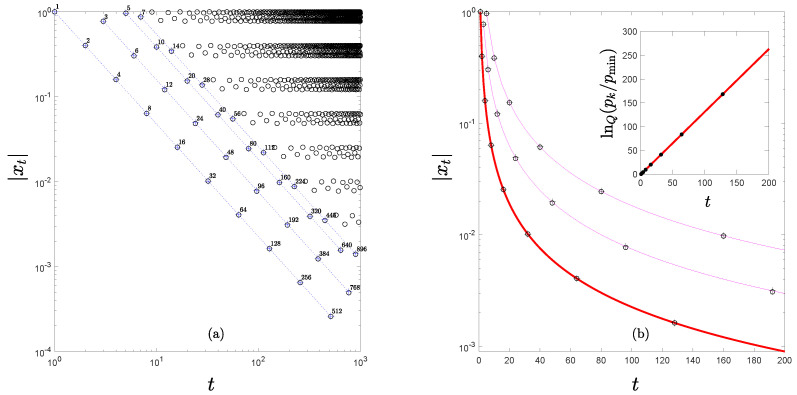
(**a**) Open circles are absolute values of trajectory positions $x_{t},t=0,1,2,\ldots$, for the logistic map $f_{\mu }\left(x\right)$ at $\mu_{\infty }$, with initial condition $x_{0}=0$, in logarithmic scales. Iteration times *t* also shown by the numbers close to the points. The lines are a guide to the eye for the diagonals described in the text. The crosses are sequences of positions computed from the RG fixed-point map trajectories in Equation (12) for the first four diagonals l=0,1,2,3 as described in the text. (**b**) The first three diagonal sequences of positions $|x_{t}|$ of the trajectory in (**a**) at iteration times $t\left(l,\tau \right)=\left(2l+1\right)2^{\tau },$ l=0,1,2 as reproduced from Equation (12) with q=1.7555. The inset shows the data for the first diagonal plotted in $\ln_{Q}p_{k}/p_{min}$ scale, $p_{k}=1/\left|x_{k}\right|$, $p_{min}=1$ and Q=0.2445. The straight line indicates (time) extensivity of the entropy $S_{Q}$.

First, we consider the case of the RG trajectories at the period-doubling transition to chaos. To illustrate this numerically we choose a specific quadratic unimodal map defined in the interval [−1,1] and with its extreme al x=0. This is $f_{\mu }\left(x\right)=1-\mu x^{2},\;-1\leq x\leq 1,\;0\leq \mu \leq 2$, with the control parameter located at $\mu =\mu_{\infty }=1.401155189092\ldots$, the value for the accumulation point of the main period-doubling cascade. In Figure 2a we show in logarithmic scales the absolute values of the trajectory positions that start at $x_{0}=0$. This principal trajectory is described in detail in Refs. [7,30,31,32]. We keep in mind that trajectories from all quadratic unimodal maps at the period-doubling transition to chaos may differ somewhat numerically from those of their RG fixed-point map but all of them approximate asymptotically that for the RG universal map for large iteration times. Here we add to (and complete) the mathematical representation [7,30,31,32] of the ‘diagonal’ structure of the principal RG trajectory. The entire RG trajectory is composed of an infinite family of position sequences that interlace consecutive iteration times. The positions of each sequence conforms with the exponential form $\alpha_{F}^{-k},k=0,1,2,\ldots$, where $\alpha_{F}=2.50290\ldots$ is the absolute value of the Feigenbaum constant, the universal scaling factor for this route to chaos (γ in Equation (10)). The slope shared by all the diagonal straight lines in Figure 2a is $-\ln\alpha_{F}/\ln2$. These two features combined lead to the expression

(20) $$x_{t}=x_{t_{w}}\exp_{q}\left(-{\left[x_{t_{w}}\right]}^{q-1}ut/t_{w}\right),$$

where $t/t_{w}=2^{k}-1,t_{w}=2l+1,l=0,1,2,\ldots ,q=1+\ln2/\ln\alpha_{F}$ and $u={\left[\left(q-1\right)x_{t_{w}}^{q-1}\right]}^{-1}$. Equation (20) can be easily corroborated to reduce to $x_{t}/x_{t_{w}}=\alpha_{F}^{-k},k=0,1,2,\ldots$, by substitution of the above definitions. We observe too that Equation (20) displays ‘waiting time’ $t_{w}$ scaling as do the ‘diagonal’ subsequences in Figure 2a.

An important mathematical conclusion about the nature of the period-doubling RG fixed-point map can be drawn from Equation (20). This is that the principal trajectory $x_{0}=0$ of the RG map can be obtained exactly from the analytical closed-form RG fixed point map for the tangent bifurcation. The argument can be extended to all other trajectories initiated at an attractor position $x_{0}\neq 0$, since any of them are obtained from the former by the removal of the (absolute value of the) positions of the principal trajectory from x=0 to the chosen $|x_{0}|>0$. See Figure 2a. Thus, each diagonal of the principal trajectory, that is, each set of subsequence positions $x_{t(l,k)},t\left(l,k\right)=\left(2l+1\right)\left(2^{k}-1\right),k=0,1,2,\ldots$, *l* fixed, in Equation (20), is also the trajectory of the RG fixed-point map $f^{*}$ in Equation (12) with z=q, $u={\left[\left(q-1\right)x_{t_{w}}^{q-1}\right]}^{-1}$ and $x_{0}=x_{t_{w}}=x_{t(l,0)}$, together with the key change of variable for the iteration times from $t/t_{w}$ in Equation (20) to τ in Equation (12) that consists of the identification k=τ. This property is shown quantitatively in Figure 2b for the first few position diagonals in Figure 2a. Furthermore, this mathematical finding can be extended to all unimodal maps of general nonlinearity other than quadratic.

**Figure 3:**
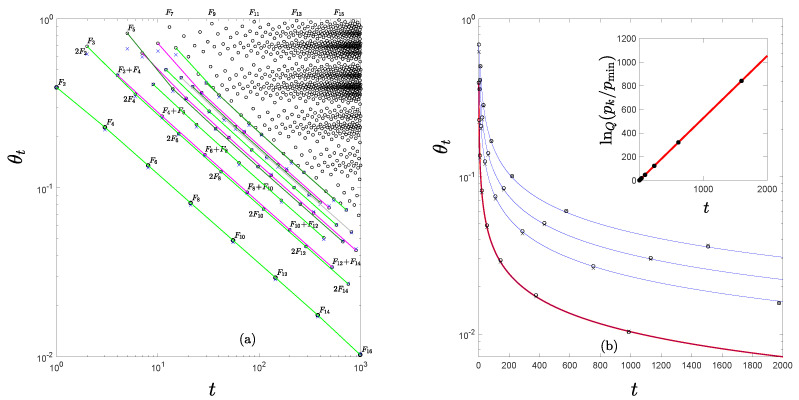
(**a**) Open circles are trajectory position values $\theta_{t},t=0,1,2,\ldots$, for the circle map $f_{\Omega }\left(\theta \right)$ at $\Omega_{\infty }^{\prime }=0.393339\ldots$, with initial condition $\theta_{0}=0$, in logarithmic scales. Iteration times *t* also shown by the numbers close to the points. The lines are a guide to the eye for the diagonals described in the text. The crosses are sequences of positions computed from the RG fixed-point map trajectories in Equation (12) for the first twelve diagonals as described in the text. (**b**) The first four leading group diagonal sequences of positions $\theta_{t}$, those with m=0 and iteration times $lF_{2n}$, of the trajectory in (**a**) as reproduced from Equation (12). See the text. The inset shows the data for the first diagonal plotted in $\ln_{Q}p_{k}/p_{min}$ scale, $p_{k}=1/\left|x_{k}\right|$, $p_{min}=1$. For the size (duration) *k* of trajectories we have used the set of Fibonacci numbers $F_{k}$ (not $F_{2k}$) in which case Q=0.051003 where Q=2−q,q=1.948997. The straight line indicates (time) extensivity of the entropy $S_{Q}$. See Ref. [11].

The RG fixed-point maps for transitions to chaos via the quasiperiodic route obey too the analytical expression Equation (11), naturally, with different universal scaling constants and rules for the specification of their intricate trajectories from those for the tangent bifurcation. To illustrate this we consider the particular case of the golden mean route to chaos often studied by means of the critical circle map $f_{\Omega }\left(\theta \right)=\theta +\Omega -{\left(2\pi \right)}^{-1}\sin2\pi \theta ,\;\mathrm{mod}\;1,0\leq \Omega \leq 1$ [24]. This particular route to chaos involves families of attractors with increasing periods $F_{n},n\to \infty$, where the $F_{n}$ are the Fibbonacci numbers. The value of Ω for their accumulation point is $\Omega_{\infty }=0.606661\ldots$, or alternatively, its mirror accumulation point $\Omega_{\infty }^{\prime }=1-\Omega_{\infty }=0.393339\ldots$ [11]. At this transition to chaos the principal trajectory, initiated at $\theta_{0}=0$, also displays a structure that decomposes into an infinite set of parallel ‘diagonals’when plotted in logarithmic scales [7,11]. These are intertwined iteration time sequences of positions that follow power laws with the same exponent value. They appear now in groups of increasing numbers, first a single diagonal, then two close diagonals, then three, and so on [7,11]. When obtained numerically from the circle map the positions for small iteration times deviate from the true power-law diagonals but for larger iteration times the positions approximate asymptotically the straight lines, indicating the mathematical form of the true RG fixed-point trajectories. In Figure 3a we show in logarithmic scales the principal trajectory of the circle map at the transition to chaos for the golden mean irrational number (for the case $\Omega_{\infty }^{\prime }$) [7,11]. The entire trajectory can be decomposed into position subsequences generated by the time subsequences, given by $t\left(l,m,n\right)=\left(l-m\right)F_{2n}+mF_{2n-2}$, each obtained by running over n=1,2,3,… for fixed values of l=1,2,3,… and m=0,1,2,…,l−1 [11]. The RG fixed-point map *q*-exponential expressions for the position subsequences are revealed if $F_{n}≃{\left(\omega_{gm}\right)}^{-n}$ is used, where $\omega_{gm}=\left(\sqrt{5}-1\right)/2$ is the reciprocal of the golden mean number [11]. For example, when this asymptotic form for $F_{n}$ is used the leading group diagonals of the principal trajectory in Figure 3a, the subsequences that lead each group, those with l>0 and m=0, i.e., positions $\theta_{t}$ with $t=lF_{2n}$, conform with the exponential form $\alpha_{gm}^{-2n},\;n=0,1,2,\ldots$, where $\alpha_{gm}=1.288575\ldots$ is the absolute value of the universal scaling factor for this route to chaos (γ in Equation (19)). The slope shared by all the diagonal straight lines in Figure 3a is $-\ln\alpha_{gm}/\ln\omega_{gm}$. These two features combined lead to the expression

(21) $$\theta_{t}=\theta_{t_{w}}\exp_{q}\left({\left[\theta_{t_{w}}\right]}^{q-1}ut/t_{w}\right),$$

where $t/t_{w}=F_{2n}-1,t_{w}=n,n=1,2,\ldots ,q=1+\ln\omega_{gm}/\ln\alpha_{gm}$ and $u={\left[\left(1-q\right)\theta_{t_{w}}^{q-1}\right]}^{-1}$. Equation (21) can be easily corroborated to reduce to $\theta_{t}/\theta_{t_{w}}=\alpha_{gm}^{-2n},n=1,2,\ldots$, by substitution of the above definitions. We observe too that Equation (21) displays also ‘waiting time’ $t_{w}$ scaling as do the‘diagonal’ subsequences in Figure 3a. We advance the conclusion that in this case, again, each diagonal of the principal trajectory is also the trajectory of the RG fixed-point map $f^{*}$ in Equation (12). That is, each set of sequence positions $\theta_{t(l,m,n)}$, with t(l,m,n),n=0,1,2,…, *l* and *m* fixed, with z=q, $u={\left[\left(1-q\right)\theta_{t_{w}}^{q-1}\right]}^{-1}$ and $x_{0}=\theta_{t_{w}}=\theta_{t(l,m,0)}$, together with the crucial change of variable for the iteration times from t(l,m,n) to τ (in Equation (12)) that consists of the identification n=τ. This property is shown quantitatively in Figure 3b for the first few position diagonals in Figure 3a.

## 4. Contraction Dimension

As we have seen the processes described by the discrete-time Landau–Ginzburg equation can be linked to the trajectories displayed by the RG fixed-point maps provided specific choices are taken that lead to the identification of its Lyapunov function with the Tsallis entropy. Since the RG fixed-point maps express the nature of the transitions to chaos along the known three routes in dissipative nonlinear dynamics these choices may capture sufficient properties for the elaboration of models for complex systems phenomena. Specific cases are mentioned in the following Section. Ensembles of trajectories with initial position sets dense across real number intervals in the domains of these maps undergo processes driven by the maps attractors that progressively confine the set of trajectories until they evolve only within the attractor positions. There is a contraction in the dimension of these ensembles, from dimension unity to a dimension less than unity as it is in the case of the period-doubling accumulation point attractor or other multifractal attractors along the quasiperiodic routes. Extreme cases correspond to tangent bifurcations where the attractor contains only a finite set of positions, one position in the case represented by the RG fixed-point map in Equation (11). In Ref. [7] we determined this dimension reduction via the introduction of a contraction dimension *Q* that relates the final probability distribution *p* on the attractor’s set of positions with the probability distribution *P* for the ensemble of initial positions,

(22) $$p_{t}=P_{t}^{Q},$$

that is,

(23) $$Q=\ln p_{t}/\ln P_{t},$$

where Q=2−q,q≤2, or Q=0 otherwise, where *q* is the *q*-deformation parameter associated with the attractor. The contraction dimension for q≤2 coincides with the change in the value of the deformed exponential parameter *q* for the algebraic inverse operation ${\left[\exp_{q}\left(x\right)\right]}^{-1}=\exp_{Q}\left(x\right)$. Thus, the dimension of the initial position space is unity for maps on an interval. Chaotic attractors do not change the dimension of this space, multifractal attractors reduce the dimension below unity, and periodic attractors, such as that at the tangent bifurcation, reduce the dimension to zero.

The algebraic inverse operation $-\ln_{Q}p_{i}=\ln_{q}p_{i}^{-1}$, q=2−Q, transforms the entropy expression in Equation (16) into

(24) $$S_{q}=\sum_{i=0}^{t}p_{i}\ln_{q}p_{i}^{-1}={\left(q-1\right)}^{-1}\left[1-\sum_{i=0}^{t}p_{i}^{q}\right],$$

so that both entropy expressions give equal values $S_{q}=S_{Q}$ provided Q=2−q. We have shown [7,8] that $S_{Q}$ in Equation (16) is obtained from the Maximum Entropy Principle with the use of the ordinary average constraint

(25) $$\sum_{i=0}^{t_{\max}}tp_{t}=\mathrm{constant}.$$

The ‘probability to a power’ expression $P_{t}^{Q}$ in Equation (22) that appears in both the *q*-statistics literature, the multifractal literature, and also in other fields, some of empirical character [33], plays here a physically meaningful role, a measure of the contraction of the set of configurations occurring in a statistical-mechanical system that signals the departure from ordinary statistics and gives rise to *q*-statistics.

Canonical partition functions $Z_{N}$ for systems with *N* degrees of freedom require a balance between growth of configuration numbers $\omega_{i}$, i=1,…,N(N) and decay of their statistical weights $w_{i}$, i=1,…,N(N), as the system size, or the number of degrees of freedom *N*, grows to infinity, N→∞. The index *i* runs over an observable like it is the energy in thermal systems. In terms of normalized weights (probabilities), $p_{i}=w_{i}/Z_{N}$,

(26) $$\sum_{i=0}^{N(N)}\omega_{i}p_{i}=1.$$

In ordinary equilibrium statistical mechanics the number of configurations $\omega_{i}$ grows exponentially with *N*, and Equation (26) remains finite, unity, in the limit N→∞ if the weights $w_{i}$ decay exponentially. This condition is reflected in the extensivity of the entropy $S_{q=1}$. When access to configurations is prevented to such an extent that $\omega_{i}$ grows only as a power law when N→∞, then the corresponding weights $w_{i}$ must decay also as a power law if Equation (26) is to remain valid and extensivity of entropy preserved. An extensive Tsallis entropy $S_{q\neq 1}$ is associated with *q*-exponential growth of $\omega_{i}$ together with *q*-exponential decay of $w_{i}$. This requires values of *q* for which the *q*-exponential grows or decays asymptotically as power laws for large argument.

As an example we retake the case of the trajectories initiated at $x_{0}<-1$ and stopped at $x_{t_{\max}}\leq -1$ of the tangent bifurcation RG fixed-point map, Equation (12). The size of the system is taken to be the size of the trajectory $N=t_{\max}$, the maximum number of configurations is identified with the absolute value of the initial position $\omega_{N}=-x_{0}$ and its normalized configuration weight is given by $p_{N}=1/\left|x_{0}\right|$. Equation (26) holds, but now configuration numbers grow and weight factors decay as *q*-exponentials that behave asymptotically as power laws.

## 5. A Collection of Research Studies and *q*-Statistics in Complex Systems

We quote briefly evidence of *q*-statistical features obtained from nonlinear dynamical models employed for specific problems in condensed matter physics and in complex systems. A more detailed description can be found in the review article “A zodiac of studies on complex systems” [18].

**Sensitivities.***q-Pesin identity at the onset of chaos and its application to diversity, a backbone model for the tree of life.* A significant property of the dynamics at the period-doubling transition to chaos is the identity between the *q*-generalized Lyapunov exponent $\lambda_{q}$ and the rate of growth of the *q*-generalized entropy $S_{q}$ [8]. This is the counterpart of the Pesin identity that states the equality of the (positive) ordinary Lyapunov exponent $\lambda_{1}$ with the Sinai–Kolmogorov entropy $K_{1}$ for chaotic attractors [24,34,35]. Actually, there is an infinite family, a spectrum, of *q*-generalized Lyapunov exponents $\lambda_{q}$, so that there is an infinite family of *q*-Pesin identities [30]. This property, that implies extensivity in iteration time, remains, conceivably, the most important exact result in *q*-statistics. The development of a first application of the *q*-Pesin identities consists of an ideal model for diversity [18], intended for biological systems or other fields where diversity is of central importance, and makes use of the Hill number, or diversity index, or effective number of species [33]. The parameter *q* defines a capacity of the diversity index to discriminate between rare and abundant species (similarly to its use in multifractals [24,34,35]).

**Partitions.***q-Partition function for sequential gap formation and its application to self-organization, including Self-Organized Criticality.* Studies [36,37] of the dynamics of ensembles of trajectories towards the attractor of the period-doubling transition to chaos, a multifractal set, led to the identification of the fraction $W_{t}$ of phase space still occupied by an ensemble of trajectories at iteration time *t* with a partition function [36,37] linked to the Tsallis statistical mechanics. Then it was realized that the same partition function arises in the construction by stages of another kind of multifractal that consists of successively halving the interval into compartments [19]. The partition function is made of compartment or subsystem configurations. Therefore, the dynamics towards the multifractal attractor at the period-doubling onset of chaos is a close analogue to a progressively constrained thermal system that initially obeys ordinary statistical mechanics [19]. The original thermal system is strongly altered and ultimately eliminated by the sequential subdivision procedure that mirrors the actions of the attractor. The emerging set of subsystem configurations implies a different and novel entropy growth process that eventually upsets the original loss and has the capability of marginally [19] locking the system into a self-organized state with characteristics of criticality, as in the so called self-organized criticality [38].

**Sums.***Distributions of sums of positions of chaotic attractors display a q-gaussian crossover from multifractal to gaussian behavior.* Examination of sums of positions of ensembles of trajectories along the band-splitting route out of chaos led to a remarkable renormalization group picture for central limit stationary distributions. The RG trivial fixed point is the ordinary normal distribution and it is reached from all chaotic attractors, while the RG nontrivial fixed point is a multifractal distribution that recapitulates the features of the period-doubling cascade and requires positioning at the transition to chaos. The flow towards the trivial fixed point displays a crossover behavior, characterized by incomplete sampling of data and therefore resembles the so-called *T*-Student distribution, that can in turn be rewritten into the form of a *q*-Gaussian distribution. For earlier studies, and a different viewpoint, of the limiting distributions of sums of positions of chaotic and marginally chaotic trajectories at both dissipative and conservative mappings see Refs. [39,40,41].

**Fluctuations.***Space & time q-exponential dominant fluctuation, a manifestation of q-entropic properties at criticality.* The employment of the Landau–Ginzburg–Wilson free energy density description for the dominant fluctuation in a model critical state revealed that *q*-statistics governs the properties of the main structural characteristic of these transient entities. The order parameter profile of the dominant fluctuation turns out to be a *q*-exponential that relates naturally to an extensive *q*-entropy. Furthermore, the time evolution of such fluctuations were put forward to be of the intermittent type, gradual growth (in amplitude or size) until collapse followed by the appearance of a new fluctuation, and so on [42,43]. This sequel would be delivered by a nonlinear map just off tangency followed by a cusp feature responsible for re-injection to the left of the bottleneck. The RG off-tangency fixed-point map for this route to chaos provides the appropriate setting for such fluctuations, where sub-occupation of phase space is an important feature.

**Glasses.***Noise-induced crossover from glassy dynamics to ordinary dynamics, the former governed by q-statistics and the latter by ordinary statistics.* A robust analogy between the dynamics of glass formation and the period-doubling transition to chaos perturbed by noise was uncovered. This makes use of the fact that the noise-induced bifurcation gap is recapitulated at such, perturbed, onset of chaos. It was shown that both processes share their main defining properties: the gradual disappearance of diffusion, the scaling law known as aging, anomalous two-step relaxation, etc. Furthermore, the ideal glass concept can be precisely represented by the attractor at the onset of chaos in the absence of noise. Variation of the amplitude of external noise (analogue to temperature) uncovers a crossover phenomenon along time evolution at the onset of chaos. Large noise amplitude corresponds to ordinary statistical mechanics, while small amplitude to *q*-statistics. Some models for traffic flow have been constructed with nonlinear dynamical elements [44], while other studies of traffic flow and arrest have found similarities with glassy dynamics [45]. A picture of a car-filled single or multiple lane road is provided by the advance of an ensemble of trajectories that fill up the positions of the attractor at the period-doubling transition to chaos. Thus the noise-perturbed onset of chaos in quadratic maps puts together the ingredients of a basic model for traffic flow and jams.

**Localizations.***q-Conductance at mobility edge of Bethe lattice model for wave scattering, the conducting, coherent, weakly-chaotic regime exhibits q-statistics.* Examination of the recursion relation for size growth of a basic wave scattering model led to a nonlinear dynamical transcription of this physical problem in terms of an iteration map with a bifurcation diagram where tangent bifurcations separate periodic (insulating) and chaotic (conducting) attractors. The sensitivity to initial conditions behaves anomalously at the localization transition and throughout the conducting phase. The ordinary Lyapunov exponent vanishes while the sensitivity obeys a *q*-exponential expression, the fingerprint of Tsallis statistics. The localization length diverges as it is the inverse of the vanishing Lyapunov exponent. The dynamics is represented by a Mobius transformation in the unit circle of the complex plane, the fixed points of which correspond to the localized states, and its ever-changing positions or phases to the extended states that display coherence. Similar reductions of degrees of freedom leading to Mobius transformations have been observed in the synchronization of arrays of oscillators [46].

**Networks.***q-Pesin equality captured by network representations of the transitions to chaos, q-statistics in Number Theory.* The exploration of an ingenious algorithm to transform time series into networks consisted of converting into networks the trajectories representative of the three routes to chaos in low-dimensional nonlinear systems [47]. This effort led to re-encounters with the generalized *q*-Pesin identities and with the underlying entropy optimization procedure associated with the renormalization group technique [48]. For the latter case it was corroborated that the RG trivial and nontrivial fixed points are extrema of a suitably defined entropy. Access to entropy was possible via the networks degree distributions. The results were positive for all cases: period-doubling and chaotic-band -splitting cascades and their common accumulation point, quasi-periodic routes to chaos, and the tangent bifurcation together with its chaotic neighborhood. At the period-doubling and the quasi-periodic transitions to chaos there appear network versions of the *q*-generalized Pesin identity. See also Ref. [47].

**Games.***q-properties at pitchfork and tangent bifurcations and at transitions to chaos have analogues in social games.* The introduction of discrete time to the replicator equation for a collection of well-known (social) games led straightforwardly into a nonlinear-dynamical extension of evolutionary game theory. The symmetric two-by-two games known as stag-hunt, hawk-dove, harmony, and prisoners’ dilemma became represented by a nonlinear iteration map in the interval with two control parameters that displays a bifurcation diagram with a rich arrangement of periodic and chaotic attractors connected by recognizable but somewhat distorted period-doubling and chaotic-band-splitting cascades, windows of periodicity, etc. The games inherit the statistical-mechanical *q*-properties described here at the evolutionary dynamics taking place at bifurcations and at transitions to chaos. One example is a game that replicates Yule’s principle (‘rich get richer’) [49], that in network language terms corresponds to ‘preferential attachment’ [50].

**Rankings.***q-statistical mechanics associated with rank distributions, universality classes and borderline contraction dimension.* By considering an existing stochastic approach for the reproduction of ranked data a formal equivalence was found with the trajectories of the RG fixed-point map for the tangent bifurcation [7]. This fact led to a nonlinear dynamical approach for rank distributions that shows similarities with universality classes in critical phenomena. This duality allows also for a clear distinction between size-rank N(k) -sizes of cities- and frequency-rank $F(k^{\prime })$ -word frequencies- distributions, as the former appears as a trajectory while the latter is a sum of positions. The frequency-rank distribution $F(k^{\prime })$ turns out to be the functional inverse of N(k). The reciprocals of N(k) provide probabilities p(k) for each *k* that lead to extensive Tsallis entropies where system size is measured by sample size [7]. A phase space contraction dimension can be defined to distinguish classes of rank distributions [7]. When q=2 (Q=2−q=0) this dimension vanishes and logarithmic corrections appear related to the known bounds for the prime numbers [18]. This is reminiscent of borderline dimensionality and its logarithmic corrections in critical phenomena.

**Measures.***Equivalence between two complex systems paradigms, edge of chaos and criticality, exhibits q-properties.* The well-known properties of families of attractors of the quadratic map were revisited as seen through the densities (or measures) of ensembles of trajectories. These probability densities were determined via the Frobenius-Perron equation and trough them a novel statistical-mechanical picture was obtained [20]. Two families of attractors were considered, the supercycles along the period-doubling cascade and the Misiurewics points along the chaotic-band-splitting cascade, together with their common accumulation point at the transition to and out of chaos. From the densities the entropies associated with these attractors were determined and, most remarkably, when the collection of entropies for the two families of attractors is viewed along the values of control parameter the familiar pattern appears of a statistical-mechanical two-phase system separated by a continuous phase transition, an equation of state containing a critical point [20]. As we have already mentioned, the transitions to chaos and the fluctuations at a critical point display the features of *q*-statistics.

Besides the review Ref. [18], the information provided here appears extended in different ways in the review articles Refs. [6,17].

## 6. Summary and Discussion

Processes that take place in systems composed of many degrees of freedom run through states with a very large number of configurations. The numbers of these configurations diminish as they reach final, simpler, states. Evolution of dissipative processes display a direction in time that involves a progressive reduction in the number of their configurations, this can be so drastic as to reduce the dimension of the configuration space. Such processes may proceed through many possible detailed paths, the set of which can be characterized with the help of probability distributions. As we have described, the most probable, or dominant, path for these systems can be quantified via macroscopic kinetic equations, an example of which is the kinetics of phase change in condensed matter. In these cases time evolution of a representative quantity, a probability density, can be computed via the Landau–Ginzburg equation, a nonlinear first-order differential equation for that quantity.

We have considered complex system processes that are better, or even necessarily, described via discrete time and indicated that the dissipative Landau–Ginzburg equation becomes in this case an iterated nonlinear map of low dimensionality. The three known routes to chaos, intermittency, period doubling and quasi-periodicity, turn out to be relevant and their RG fixed-point maps take front roles in the modelling of these problems. As we have seen, at the transitions to chaos phase-space contraction dimension is manifest, from that of real number intervals to those of multifractal or finite sets of positions. We have shown that these universal maps and their trajectories all obey the same analytic, closed-form expressions. The simplest is the known [9,24]) RG fixed-point map for the tangent bifurcation, but the original contribution described here is that the trajectories of the other two fixed-point maps can be obtained from the former with the use of specific rules that define sets of time iteration changes of variable. Most significant is the fact that trajectories of the fixed-point maps that describe time evolution obey scale invariance as observed in many real complex system behaviors.

We have shown that the RG fixed-point maps can be seen as discrete-time versions of the Landau–Ginzburg kinetic equation and that they are associated with a Lyapunov function given by the Tsallis entropy. The monotonic iteration time evolution of this function obeys *q*-statistics and displays *q*-exponential partition function configuration weights. We have also briefly referred to an interelated set of complex system research lines that make use of models based on nonlinear dynamics of low dimensionality. These studies capture the features and provide predictions for central complex system questions, such as, diversity, self-organization, stationary distributions, critical fluctuations, glassy dynamics, localization transitions, scale invariant networks, evolutionary game theory, ranked data distributions, and paradigm equivalence of edge of chaos and criticality. Other research topics involve, biology and urban allometry and nested systems [18].

The processes described by the Landau–Ginzburg equation correspond to those occurring in high-dimensional systems, originally in condensed matter physics, in magnets, fluids, liquid crystals, superconductors, etc.; systems in the thermodynamic limit, with infinite numbers of degrees of freedom. The starting point of the derivation of the MKR Equation (3) is an equation for all the degrees of freedom of a high-dimensional system [21,22]. The final equation is a first-order differential equation (or a one-dimensional iteration map) for the same high-dimensional system. The robustness of our results in high-dimensional settings is similar to the robustness of the results provided by the Landau–Ginzburg equation (or the Cahn–Hilliard equation, or the MKR equation, or those for other related statistical-mechanical approaches). The occurrence of *q*-exponentials in models for high-dimensional complex systems is the counterpart of the occurrence of exponential Boltzmann weights in conventional high-dimensional systems. The general validity of the Tsallis statistics to describe processes in these systems rests on a drastic reduction of accessible configurations, a reduction into a subset of zero measure with respect to the initial one. A vanishing measure of available configurations occurring as a result of such a process in a high-dimensional system results in a final subset of configurations that is still high dimensional. This is projected into the description provided by a discrete-time Landau–Ginzburg equation as a one-dimensional (real number) set of initial conditions that ends up as a multifractal or finite set of final conditions.

## Data Availability

Not applicable.
